# Two-dimensional linkage analyses of rheumatoid arthritis

**DOI:** 10.1186/1753-6561-1-s1-s68

**Published:** 2007-12-18

**Authors:** Nandita Mukhopadhyay, Indrani Halder, Samsiddhi Bhattacharjee, Daniel E Weeks

**Affiliations:** 1 Department of Human Genetics, Graduate School of Public Health, University of Pittsburgh, A300 Crabtree Hall, 130 DeSoto Street, Pittsburgh, Pennsylvania 15261, USA; 2 Behavioral Physiology Laboratory, Cardiovascular Behavioral Medicine Program, University of Pittsburgh, Pittsburgh, Pennsylvania 15261, USA; 3 Department of Biostatistics, Graduate School of Public Health, 130 DeSoto Street, University of Pittsburgh, Pittsburgh, Pennsylvania 15261, USA

## Abstract

Rheumatoid arthritis (RA) is a multifactorial disease with complex genetic etiology, about which little is known. Here, we apply a two-stage procedure in which a quick first-stage analysis was used to narrow down targets for a more thorough and detailed testing for gene × gene interaction. Potentially interesting regions were first identified by testing for major gene effects using non-parametric linkage methods. To select regions of interest, we first tested for linkage to three different RA-related traits one at a time: RA affection status and the quantitative phenotypes rheumatoid factor IgM and anti-cyclic citrullinated peptide levels. These linkage analyses identified regions on chromosomes 3, 5, 6, 8, 16, 18, 19, and 20. We subsequently analyzed the selected regions in a pairwise manner to detect gene × gene interactions influencing RA using a recently developed two-dimensional linkage method. We found evidence of interacting loci on chromosomes 5, 6, and 18.

## Background

Rheumatoid arthritis (RA) is a complex inflammatory disease primarily affecting the joints. Research on the genetic basis of RA has identified several loci with potentially significant effects, including *HLA-DRB*, *PTPN8*, *NFKBIL1*, *RUNX1*, and *SLC22A4*.

Whole-genome scans typically focus on identifying single genes with major effects and often lack power to detect linkage to sets of interacting genes. A two-dimensional genome scan has recently been used to simultaneously detect linkage to two interacting genomic regions in the study of hypertension by Bell et al. [1].

In this study, we attempt to identify gene × gene interactions influencing RA by employing a two-stage linkage analysis procedure. In the first step, one-dimensional linkage analyses of each of the three phenotypes-RA status, rheumatoid factor IgM and anti-cyclic citrullinated peptide levels-were carried out using genome spanning microsatellite markers. We then selected single-nucleotide polymorphims (SNPs) (from a panel of 5 K SNPs), which were located in the chromosomal regions showing elevated linkage signals for any of these three traits, which were analyzed in a pair-wise manner for linkage to RA in the two-dimensional (2D) genome scan step. We present the results of the 2D genome scan on 5-cM regions flanking each marker that showed a one-dimensional (1D) trait-specific LOD score above 1.5. Because traditional LOD score thresholds are not applicable to a 2D genome scan, we determined significance levels for the 2D genome scan results by comparing them against their empirical distributions (obtained using simulated sets of unlinked markers).

## Subjects and methods

### Phenotypes

The genotype and phenotype data are from the North American Rheumatoid Arthritis Consortium (NARAC) [2]. We analyzed three traits separately, including two quantitative traits known to co-occur strongly with rheumatoid arthritis [3], namely, rheumatoid factor IgM (denoted as RF in the rest of this manuscript), and anti-cyclic citrullinated peptide levels (denoted anti-CCP). The third trait was RA affection status, coded as 0: unknown, 1: unaffected, and 2: affected. The quantitative phenotypes were not transformed; instead we chose to use quantitative trait locus (QTL) mapping statistics, which are robust to distributional assumptions.

### Pedigrees and genotype data

Our data consisted of 472 multiplex pedigrees containing 1164 genotyped persons, 1076 phenotyped for the anti-CCP trait, and 1094 phenotyped for the RF trait. We used all 402 autosomal microsatellite markers for the 1D linkage analysis of the three traits. For two-locus analysis, we selected a subset of SNPs from the 5 K SNP scan of the NARAC data [4] on chromosomes 5, 6, 8, 16, 19, and 20. Allele frequencies for microsatellite markers were estimated from the observed genotype data using Mega2 [5]. SNP allele frequencies were obtained from Illumina's web site [6]. These were in good agreement with frequencies estimated from the NARAC families (results not shown). Genetic positions for SNPs were ascertained from the Linkage IVb genetic maps from Illumina's website. All map distances are in Kosambi centimorgans.

### Non-parametric linkage on microsatellite scan

Merlin version 1.01 [7] was used to perform linkage analysis on the RA affection status using the NPL-ALL statistic, using all genotyped individuals. For the two quantitative phenotypes, we used an equally weighted sum of the Haseman-Elston trait regression statistic and the mean allele-sharing test statistic to detect linkage, both implemented in SIBPAL, as part of S.A.G.E. version 5.2 [8]. In the Haseman-Elston procedure, the cross-product of trait sums and trait differences is regressed on identity-by-descent (IBD) allele-sharing, whereas the mean-allele sharing test simply compares the observed proportion of alleles shared IBD against expected proportions. Haseman-Elston trait regression analysis on the two quantitative traits used 635 sibling pairs.

### Gene × gene interactions on SNP scan

A 2D genome scan was performed for RA affection status to test for gene × gene interactions using the Merloc program [1]. Merloc models the recurrence risk ratio of disease as a function of variances and covariances for a putative disease locus pair. The variances and covariances can be expressed as functions of the two-locus IBD states [9,10]. Merloc then searches over several combinations of variance components corresponding to several different two-locus interaction models: single gene at either locus, additive (no epistasis), multiplicative (epistasis level = 1.0), epistatic (epistasis > 1), and a general model containing all additive and dominance variance components, reporting the maximum LOD score (MLS) for each model and pair of loci.

For the 2D linkage analysis, we used 746 phenotyped pedigrees for whom 5 K SNP scan data was also available. These were divided into 1724 nuclear families because Merloc currently only handles sibship data); subsequently uninformative pedigrees were trimmed using Merlin, leaving 724 informative nuclear families with 3053 individuals.

### Selection of SNPs for two-locus analysis

We selected a subset of loci from the 5 K SNP data, which were located in the significant regions identified from the microsatellite scans. In this step, each marker locus from one region is paired with each marker locus from a second region in turn, and analyzed as a pair. For selecting the subset of SNPs we first identified microsatellite markers with LOD scores >1.5 (which translates to a theoretical marginal significance level of ~0.004) and then selected SNPs within a 10-cM region flanking these markers. Where two or more loci with LOD score >1.5 occurred in close proximity, we selected a single region of 10 cM centered on the marker with highest LOD score. In instances in which more than two sites were included on the same chromosome, we confirmed there was reasonable separation between the regions. Such a constraint on the distance between regions on the same chromosome was necessary because the 2D genome scan method assumes locus pairs are unlinked. The 11 separate regions selected finally are shown in Table 1.

**Table 1:** Non-parametric linkage results on RA, and quantitative linkage analysis on RF and anti-CCP

Chr	Region (cM)	Marker	Position	LOD	Trait(s)	No. of SNPs	LD (*r*^2^)
3	151.86–161.25	F45a	155.5	4.90	RF, anti-CCP	8	0.025
5	14.69–23.45	F25a	19.0	1.54	RF	6	0.014
6	30.33–38.32	T39a	34.23	11.13	RA*, RF, anti-CCP	4	0.100
	47.06–67.19	D6S291	49.5	13.95	RA*, RF, anti-CCP	10	0.100
	124.80–133.19	H10a	128.93	1.96	RF	7	0.100
8	3.40–12.29	F36b	8.34	1.71	RF, anti-CCP*	7	0.012
16	39.95–48.71	F10a	43.89	2.06	RA	6	0.014
	95.48–105.28	H3c	100.39	1.53	RA	7	0.014
18	75.58–84.57	H43a	80.41	2.04	RA*, anti-CCP	8	0.200
	110.96–119.98	F12a	115.89	1.62	anti-CCP	7	0.200
19	83.91–91.09	F31a	87.66	1.74	RF*, anti-CCP	5	0.025
20	5.81	F26a	2.13	1.71	anti-CCP	1	NA

In the trait column, where more than two traits are listed, the asterisk (*) marks the trait with the higher LOD score, which is listed in the LOD score column. If no trait is indicated with an asterisk, all had the same LOD score value.

Preliminary analysis indicated that similar results are obtained with closely spaced SNPs using the two-locus method (data not shown), which could possibly be due to LD between closely spaced SNPs within a region. So, we estimated LD between markers within each of the 11 regions identified, but not across region pairs because these were selected to be reasonably distant from each other. LD was estimated using H-clust [11] on a subset of the individuals, selecting one genotyped individual at random from each pedigree. The maximum pairwise LD value for each region is shown in Table 1. Most SNPs in the selected regions had low LD between them. Although all SNPs were acceptable based on LD, we further trimmed our SNP set by including markers placed ~1 cM apart from each other within a region in order to reduce computational complexity.

### Empirical estimation of significance

We simulated 1000 replicates of genotype data for 24 locus pairs contained within the 14.69–23.45 cM region on chromosome 5 and the 30.33–38.32 cM region on chromosome 6. Unlinked genotype data for SNPs were simulated using the program Simulate by Terwilliger et al. [12], while maintaining the pedigree structures and phenotypes of the NARAC families. Model-specific empirical LOD score distributions were obtained by pooling LOD scores on the 24 locus pairs for each two-locus model. We then compared the 2D genome scan results to the upper 0.1 percentile values of the appropriate model-specific distribution to assess empirical significance.

## Results

### Non-parametric linkage analysis of RA affection status

We observed LOD scores >1.5 on chromosomes 6, 16, and 18 (Table 1). A 60-cM region between microsatellite markers T1c and H13c on chromosome 6 demonstrated highly elevated LOD scores of up to 13.9. Elevated LOD scores were also observed around the F10a microsatellite marker (chromosome 16), and near the H43a microsatellite marker (chromosome 18).

### Quantitative analysis of RF and anti-CCP

For the RF trait, elevated LOD scores were obtained on chromosomes 3, 5, 6, and 8, with the highest score obtained on chromosome 6 (which is also the site of HLA locus (6p21.3)). Chromosomes 3, 6, 8, 18, 19, and 20 had elevated linkage signals for the anti-CCP trait. Both RF and anti-CCP showed a strong linkage signal on chromosome 3 as well.

### Two-locus analysis results

**Figure 1:**
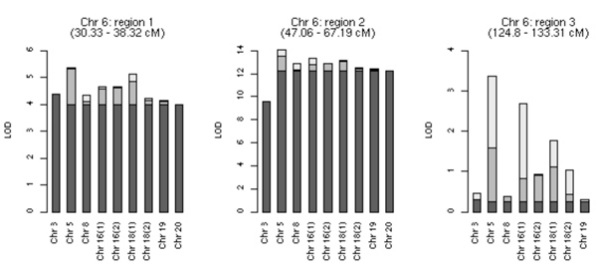
**Single-locus contributions of chromosome 6 SNPs vs. other SNPs**. Dark grey, single-locus LOD scores of the chromosome 6 SNP; light gray, single-locus LOD scores of the other SNP; unshaded, the interaction contribution. Numbers in parentheses on the X-axis indicate separate regions on a single chromosome.

Figure 1 contains two-locus results of SNPs on chromosome 6 with SNPs on other chromosomes. Because the two-locus analysis estimates interaction effects after fixing the single-locus LOD scores for each of the two loci, comparing the remainder of the general LOD score to the individual contributions provides us with a measure of gene × gene interaction effects. The total height of each bar represents the maximum two-locus LOD score obtained for the general model, with the dark shaded area representing the marginal score for the chromosome 6 SNP, the lightly shaded area being the marginal contribution of the second SNP, and unshaded area being the remaining contribution, including two-locus interaction effects. All analyses involving region 2 on chromosome 6 showed highly elevated LOD scores in the two-locus analysis, and most were due to the marginal single-locus contributions of SNPs on chromosome 6. The chromosome 5-chromosome 6:region 1 locus pairs have marginal contributions from both loci, as well as a total additive effect. In region 3 of chromosome 6 the general model LOD scores appear to be larger than either single-locus LOD score. The actual LOD score values are given in rows two and three of Table 2.

**Table 2:** Locus-pairs with two-locus maximum LOD scores greater than the maximum marginal single-locus LOD scores

Locus1:chr	Locus2:chr	SL1	SL2	ADD	MULT	EPI	GEN
rs755763:**3^a^**	rs2066272:**6**	0.0	4.37	**4.37**	**4.37**	**4.37**	**4.37**
rs2173946:**5**	rs2066272:**6**	1.33	3.98	**5.24**	**5.32**	**5.33**	**5.35**
rs3846569:**5**	rs1407529:**6**	1.33	0.25	1.49	1.58	**3.25**	**3.37**
rs246765:**5**	rs1792737:**18**	1.33	0.87	2.10	2.21	**2.73**	**2.74**
rs2066272:**6**	rs1478959:**8**	3.98	0.14	**4.06**	**4.12**	**4.32**	**4.33**

^a^Bolding indicates two-locus LODs greater than the 99.9% empirical threshold.

Table 2 lists pairs of regions, for which one or more of the two-locus LOD scores, were found to be above the respective 99.9% empirical thresholds observed from our simulations. The first two columns contain SNP names and chromosome numbers. The other five columns contain maximum LOD scores obtained for each separate model, i) single-locus MLS at locus 1 (SL1), ii) single-locus MLS at locus 2 (SL2), two-locus additive MLS, i.e., epistasis parameter = 0.0 (ADD), two-locus multiplicative MLS, i.e., epistasis parameter 1.0, two-locus epistatic model with epistasis parameter allowed to vary above 1.0 (EPI), and the completely unrestricted two-locus MLS (GEN).

Evidence of interaction can be inferred in this table by comparing the maximum of the MLS over all locus-pairs within a pair of regions, then comparing it in turn to the maximum of the MLS of the respective single-locus models within those two regions. For example, in the second row of Table 1, the SNPs rs2173946 on chromosome 5p13.5 and rs2066272 on chromosome 6p22.3 have a two-locus MLS that is somewhat larger than either of the single-locus MLSs, and the interaction could be either additive or multiplicative. In comparison, the third row shows a possible epistatic interaction (the EPI MLS being appreciably larger than the multiplicative and additive two-locus MLSs). Several of the locus pairs containing the chromosome 6 SNP rs2066272 that had LOD scores above the threshold value had similar scores to the last pair reported in Table 2.

### Empirical *p*-values

We pooled the LOD scores from all 24,000 locus pairs from the 1000 replicates to derive an empirical distribution of the two-locus LOD scores. The threshold values observed at 1% and 0.5% significance levels for the additive, multiplicative, and epistatic models are all around 1.85 and 2.07, respectively. The general model has higher threshold values than the more restricted models, 1.93 and 2.13, respectively. Single-locus threshold values were close to their expected values, 1.28 and 1.68, respectively. Threshold values for 0.1 level, used to assess significance of the 2D genome scan results, were all similar ranging from 2.42 for the single-locus model to 2.57 for the general model.

## Discussion

The bulk of the NARAC data consists of affected sib pairs, and because IgM and anti-CCP are both strongly related to RA, family members are expected to be phenotypically concordant. It has been suggested (E. Feingold and colleagues, unpublished data) that regressing traits on IBD data alone has very low power to detect linkage in the presence of highly concordant relative pairs, and, in such a situation, the allele-sharing statistic contains most of the information regarding linkage. Variance-components analysis was not appropriate for this data because both quantitative traits show a highly non-normal distribution within the NARAC pedigrees. The method proposed by Sham et al. [13], which regresses allele-sharing on the trait, was also not applicable: it requires good estimates of population mean, variance and heritability, which were unavailable.

We chose not to include two-locus analysis on syntenic loci. It has been previously noted by Bell et al. [1] that the recombination fraction between these loci affects two-locus MLS as well. In the course of our analysis a few closely situated syntenic regions produced highly erratic MLS values (results not shown).

The locus pairs within each simulation replicate are correlated, so the effective number of independent observations is smaller than 24,000. Thus, our simulation size may not be large enough to accurately estimate the extreme quantiles. Moreover, SNP genotypes have limited variation within the replicates, which further imposes limits on the range of possible LOD scores that can be obtained.

## Conclusion

Searching for gene × gene interactions over a dense genome-scan data poses many difficulties, such as demanding computations, and may result in high false-positive rates. Thus, we decided to limit the number of SNPs based on certain predefined criteria. Rather than relying on the affection status alone, we used linkage of two quantitative traits for selecting the regions of interest. Subsequent 2D genome scanning showed promising evidence of linkage as well as gene × gene interactions between chromosomal regions. Interestingly, these interacting regions have also recently been identified as being linked with RF and anti-CCP on the same data set [14] and this report further establishes evidence of gene × gene interaction between these regions. However, as shown in from Figure 1, the bulk of the two-locus LOD scores involving chromosome 6 loci and other regions can be attributed to chromosome 6 loci. It is likely from our results that detecting interaction between two genes may be difficult where one of the genes has a strong marginal effect.

## Competing interests

The author(s) declare that they have no competing interests.
